# Effect of *Echinophora platyloba* DC. essential oil and lycopene on the stability of pasteurized cream obtained from cow milk

**Published:** 2016-06-15

**Authors:** Ali Ehsani, Mohammad Hashemi, Nima Hosseini Jazani, Javad Aliakbarlu, Sajad Shokri, Seyedeh Samane Naghibi

**Affiliations:** 1 *Department of Food Hygiene and Quality Control, Faculty of Veterinary Medicine, Urmia University, Urmia, Iran; *; 2 *Department of Food Science and Technology, Faculty of Nutrition, Tabriz University of Medical Sciences, Tabriz, Iran; *; 3 *Department of Nutrition, Faculty of Medicine, Mashhad University of Medical Sciences, Mashhad, Iran; *; 4 *Food and Beverages Safety Research Center, Urmia University of Medical Sciences, Urmia, Iran.*

**Keywords:** *Echinophora Platyloba*, Lycopene, Pasteurized cream, Shelf-life

## Abstract

The present study was carried out to enhance shelf life of pasteurized cream using *Echinophora platyloba* essential oil (EEO) and lycopene. For this purpose, two concentrations of EEO (0.10% and 0.50%) and lycopene (20 and 50 ppm) alone and together as combinations were added in pasteurized creams and analyzed for microbial characteristics, sensorial properties and lipid stability during storage at 4 ˚C and 25 ˚C for 14 days. Results of microbial and chemical analyses of experimental pasteurized creams showed that pasteurized creams treated with combinations of the EEO and lycopene in their higher concentrations had the best microbial and chemical properties and the most stability than control during storage (*p* < 0.05). Results of sensorial evaluation demonstrated that all treatments had favorable overall acceptability, even though, the best sensorial properties were observed in creams with combinations of EEO and lycopene in their lower concentrations. Therefore, based on the results of the present study, application of EEO and lycopene as natural preservatives is especially recommend in high fat dairy products such as butter and cream.

## Introduction

Cream is a selective milk concentrate containing an elevated level of milk fat globules dispersed in a continuous phase of skim milk.^1^ Definition for heat treatments of creams is heated to a temperature not less than 72 ˚C and retained at that temperature for not less than 15 sec or heated to any other temperature for such other period of time as has equivalent effect for the elimination of vegetative pathogenic organisms in the cream. Such pasteurized creams must be cooled as soon as practicable after pasteurization and show a negative reaction to the phosphatase test.^2^

Since cream is a product with high fat content, it is more prone to lipolysis than to proteolytic spoilage caused by the thermo-stable enzymes of psychrotrophics.^3^ Bacteriological defects usually do not arise at low temperature, but chemical defects occur due to auto-oxidation. Oxidative rancidity is the major problem arising with high fat dairy products during storage.^4^ Lipid oxidation is a major quality problem in the processing and storage of oils, fats and fat-containing foods affecting many characteristics of them such as color, flavor, texture and nutritional value.^5^ Hydro-peroxides are initial oxidation products which accumulate and may subsequently break down to form lower molecular weight compounds such as alcohols, aldehydes, free fatty acid (FFA) and ketones, leading to auto-oxidative rancidity.^6^

The process of fat oxidation can be prevented by adding natural or synthetic antioxidant substances. However, it is well known that the application of synthetic antioxidants such as butylated hydroxy anisole, butylated hydroxy toluene, and butyl hydroquinone in food could cause hepatic damages, toxicities, and cancer in laboratory animals.^7^ On the other hand, increasing trend in food industry, driven by consumer concerns, has been a shift from synthetic to natural antioxidants for inhibiting the development of oxidative rancidity in fat-containing foodstuffs.^8^

Lycopene, the pigment principally responsible for the characteristic deep-red color of ripe tomato fruits and tomato products, has received much attention in recent years. It has many distinct and unique biological properties due to its acyclic structure, large array of conjugated double bonds and extreme hydrophobicity.^9^ This compound is a fat soluble carotenoid with at least twice the antioxidant capacity of β-carotene and 10 times higher than that of R-tocopherol, which makes its presence in foods of considerable interest. Tomato skin is a rich source of lycopene, as it contains five times more lycopene than the whole tomato pulp.^10^ Tomato lycopene has all the advantages to make it an excellent natural food additive including stability to heat and extreme pH encountered during food processing, effectiveness even in low concentrations and covering a range of colors from yellow through orange to deep-red without having off-flavors.^11^

*Echinophora platyloba* DC. is a perennial plant, of the most fascinating species and is well known for its favorable aromatic property. Fresh and dried aerial parts of this species are used as a seasoning and anti-mold agent in pickled cauliflower, gherkin, and native dairy products like cheese, yoghurt and doogh in west and northwest of Iran.^7^

To the authors’ best knowledge, no comprehensive work has been carried out on anti-oxidative or antimicrobial activities of *E. platyloba* DC. essential oil (EEO) in any food model. Also, effects of lycopene have not been researched on cream properties. Therefore, this study aimed to assess the effects of EEO and lycopene on microbial and sensory properties as well as chemical stability of pasteurized cream during storage.

## Materials and Methods

The aerial parts of *Echinophora platyloba *DC. were collected during flowering stage (June 10^th^ to August 15^th^, 2010) from Maragheh city (northwest of Iran), and identified by the Herbarium of West-Azerbaijan Agricultural and Natural Resource Center, Urmia, Iran (Voucher specimen no.: 6502). Then, it was dried and ground into powder. The prepared powder was kept in tight containers protected completely from light. Pasteurized cream (30.00% fat) was provided by Pegah dairy industries Urmia, Iran. Lycopene, butylated hydroxyl toluene (BHT) and thiobarbituric acid was obtained from Sigma-Aldrich chemical Co. (St. Louis, USA). Analytical grade methanol, butanol, high performance liquid chromatography (HPLC) chloroform, diethyl ether, glacial acetic acid, sodium thiosulphate, sodium hydroxide, potassium iodide and all cultures media were purchased from Merck (Darmstadt, Germany).

**Isolation and analysis of essential oil.** *Echinophora platyloba *DC. essential oil was provided according to the method recommended by the European Pharmacopia,^12^ and chemical composition of oil was analyzed by gas chromatography/ mass spectrometry (Agilent 6890 gas chromatograph equipped with an Agilent 5973 mass-selective detector; Agilent, Swindon, UK) as described by authors previously.^13^

**Sample preparations.** Pasteurized cream obtained from fresh cow milk was divided into sterile glass containers and two concentrations of filtered EEO (0.10% and 0.50%) and lycopene (20 and 50 ppm) alone and together as combinations were added to the creams according to following treatments: 1) 0.10% EEO (E_1_), 2) 0.50% EEO (E_2_), 3) 20 ppm lycopene (L_1_), 4) 50 ppm lycopene (L_2_), 5) 0.10% EEO + 20 ppm lycopene (E_1_L_1_), 6) 0.10% EEO + 50 ppm lycopene (E_1_L_2_), 7) 0.50% EEO + 20 ppm lycopene (E_2_L_1_), 8) 0.50% EEO + 50 ppm lycopene (E_2_L_2_). Pasteurized cream samples with 0.02% BHT and with no additives (essential oil, lycopene or BHT) as control were prepared as well. The samples were stored in refrigerator at 4 ˚C and at room temperature (25 ˚C) in the dark for 14 days and analyzed periodically on days 0, 4, 7, 10 and 14 for chemical and microbiological assessments and sensory analysis. Separate samples were prepared for measurements during storage and all measurements were performed in triplicate.

**Microbiological analysis. **For microbial analysis 1 g of samples was transferred aseptically to sterile tubes containing 9 mL buffered sterile peptone water (0.10%) and homogenized. Decimal dilutions were prepared in sterile peptone water and plated on media.^5^^,^^14^ Psychrotrophic bacteria were counted using king agar medium, after incubation for 48 hr at 21 ˚C. *Pseudomonas *spp. were enumerated on cetrimide agar following incubation for 48 hr at 37˚C. After incubation at 37 ˚C for 48 hr, total aerobic mesophilic bacteria (TAMB) counts were also made on nutrient agar.^14^ Microbiological data are logarithm of the number of colony-forming units per gram (log CFU g^-1^).

**Peroxide value. **Peroxide value (PV) was measured using the procedure proposed by Egan *et al.*^15^ A portion (0.10 g) of extracted oil of cream samples was mixed with 25 mL solution of acetic acid and chloroform (ratio 3:2, v/v), and 1 mL of saturated potassium iodide was then added. The mixtureswere kept in a dark place for about 10 min, and then 20 mL of distilled water and 1 mL of freshly prepared 1.50% starch were added to the samples. After shaking, the samples and an oil free sample were titrated with 0.01 N sodium thiosulphate until the blue color disappeared. The peroxide values were expressed as milli-equivalents of peroxide oxygen per kg of lipid (mEq kg^-1^).

**Thiobarbituric acid reactive substances (TBARS). **The TBARS were determined according to Abuzaytoun and Shahidi*.*^16^ Extracted oil of cream samples (0.20 g),^17^ were mixed with 25 mL of butanol. This mixture (5 mL) was transferred to a dry test tube and 5 mL of TBA reagent (0.20 g of TBA were dissolved in 100 mL of butanol) were added to the same test tube. The mixture was well mixed and heated in a boiling water bath (Fan Azma Gostar, Tehran, Iran) for 2 hr and the intensity of the resultant colored mixture was measured at 532 nm after cooling under running water. Butanol (5 mL) with TBA reagent (5 mL) were used as the blank and TBARS values were expressed as mg malondialdehyde (MDA) per kg of sample.

**Free fatty acids** *.* Free fatty acids of samples were detected using Egan *et al*.’s method.^15^ Solution of ethanol and diethyl ether (50 mL; ratio 1:1, v/v) were added to 0.20 g of extracted oil of cream samples,^17^ and the mixture was titrated with 0.10 N NaOH in the presence of phenol phetaleine until the pink color appeared and lasted for at least 30 sec. Results were expressed as percent (%) oleic acid.

**Sensory analysis.** The samples were evaluated using a 9-point hedonic scale (1 to 3.9, 4 to 6.9 and 7 to 9 indicated the limit of unacceptability, moderate acceptability and high acceptability) by a semi-trained panel. Panel members (nine people) were instructed about the product and its characteristics and were selected based on their performance in initial evaluation trials from the laboratory staff and PhD students of food hygiene, Department of Food Hygiene and Quality Control, Faculty of Veterinary Medicine, Urmia University, Urmia, Iran. Sensory attributes of the samples were appearance (visual appeal on sight), color (adequacy of hue and uniformity), aroma (olfactory feeling on inhaling the head space volatiles), taste (response of taste bud on masticating), texture (force to chew and consistency on melting), and overall acceptability (likeness as compared to commercial product).^18^

**Statistical analysis.** The results were presented as mean ± SD. Statistical analysis of the data was made using the analysis of variance of the SPSS (version 18.0; IBM, Armonk, USA). Means with a significant difference (*p *< 0.05) were compared by Duncan’s post hoc test.

## Results

**Fig. 1:**
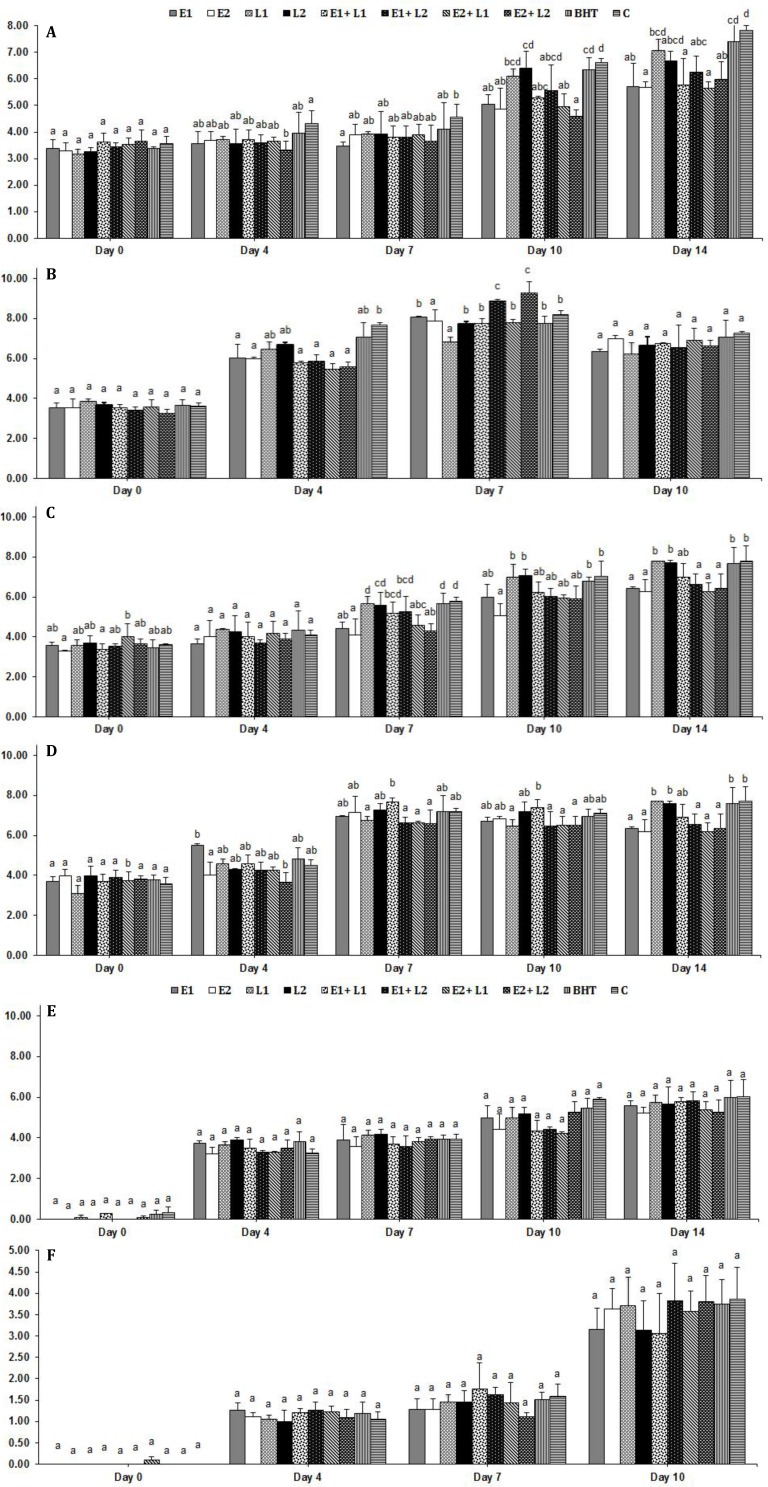
Changes in microbial counts (log CFU per g) of pasteurized creams treated by *Echinophora platyloba* DC. essential oil and lycopene during storage. Data are presented as mean ± standard deviation. **A)** Total aerobic mesophilic bacteria count at 4 ˚C, **B)** Total aerobic mesophilic bacteria count at 25 ˚C, **C)** Psychrotrophic count at 4 ˚C, **D)** Psychrotrophic count at 25˚C, **E)** *Pseudomonas* Spp. count at 4˚C and **F)** *Pseudomonas* Spp. count at 25˚C. Same letters indicate no significant differences (*p > *0.05).

**Microbial changes.** All microbial changes are shown in Figure 1. Total aerobic mesophilic bacteria count increased in all of the samples during storage. The highest TAMB counts were determined in the control and BHT samples and the lowest were determined in samples containing EEO especially high level of the oil (0.50%) with a significant difference with control (*p* < 0.05). As are shown, samples with high concentration of EEO (0.50%) had the lowest psychrotrophic changes and significant difference with control at the end of the storage period (*p* < 0.05). *Pseudomonas *spp. count increased in all of the samples during storage but there was no significant difference (*p* < 0.05) between treatments. Microbial counts of all samples stored at 25 ˚C were not analyzed on 14^th^ day of storage period because of cream putrefaction.

**Lipid damage. **Effect of treatments on PV of samples stored at 4 ˚C and 25 ˚C are presented in Table 1. For both 4˚C and 25˚C stored samples, the initial amount of PV values was not significantly different between the samples but at the end of storage period, PV of all treated samples were significantly lower than control. Samples containing high concentration of EEO and lycopene (E_2_L_2_) as well as samples containing BHT had the lowest PV. 

All treated pasteurized creams had approximately similar FFA content at the time of preparation, which increased to 3.57 - 5.10% (g oleic acid per g fat of sample) in samples stored at 4 ˚C and to 8.22-12.12 (g oleic acid per g fat of sample) in samples stored at 25 ˚C at the end of storage time (Table 2). Besides BHT, samples with combined application of EEO and lycopene had lower FFA values with the lowest FFA values for 0.50% EEO + 50 ppm lycopene (E_2_L_2_) samples.

E_1_: 0.10% EEO, E_2_: 0.50% EEO, L_1_: 20 ppm lycopene, L_2_: 50 ppm lycopene, BHT: 0.02% BHT, C: Control.

**Table 1:** Peroxide values (mEq kg^-1^) of pasteurized creams treated by *Echinophora platyloba *DC. essential oil and lycopene during storage. Data are presented as mean ± standard deviation

	**Treatments**	** Day 0**	** Day 4**	** Day 7**	** Day 10**	** Day 14**
***4*** ***˚C***	**E1**	22.92 ± 0.40^aA^	45.56 ± 2.98^aB^	56.42 ± 1.37^bC^	67.13 ± 1.42^bD^	71.77 ± 0.15^bE^
	**E2**	23.53 ± 0.11^aA^	44.89 ± 1.02^aB^	55.95 ± 1.34^bC^	68.54 ± 0.51^bD^	71.01 ± 1.13^bE^
	**L1**	22.43 ± 2.15^aA^	36.57 ± 1.70^bB^	50.20 ± 1.41^cC^	59.42 ± 4.51^cD^	61.91 ± 1.69^cD^
	**L2**	22.80 ± 3.04^aA^	30.35 ± 1.06^bcB^	40.65 ± 0.77^dC^	48.45 ± 4.62^dD^	51.00 ± 1.98^dD^
	**E1+L1**	22.49 ± 2.67^aA^	36.08 ± 1.16^bB^	48.39 ± 1.65^cC^	59.27 ± 1.41^cD^	62.24 ± 0.50^cD^
	**E1+L2**	23.10 ± 2.68^aA^	27.24 ± 2.79^cA^	36.27 ± 1.16^eB^	43.06 ± 1.4^deC^	46.76 ± 0.23^eC^
	**E2+L1**	23.13 ± 1.17^aA^	35.28 ± 4.00^bB^	46.72 ± 1.33^cC^	58.26 ± 0.49^cD^	60.00 ± 0.99^cD^
	**E2+L2**	23.06 ± 3.67^aA^	26.13 ± 2.84^cA^	42.66 ± 0.42^eB^	39.63 ± 2.08^eC^	40.01 ± 0.27^fC^
	**BHT**	22.86 ± 2.30^aA^	26.18 ± 1.23^cAB^	33.2 ± 3.39^eBC^	38.99 ± 5.41^eC^	39.75 ± 1.06^fC^
	**C**	22.13 ± 2.06^aA^	48.50 ± 0.54^aB^	63.31 ± 1.43^aC^	77.44 ± 3.06^aD^	81.20 ± 0.86^aD^
***25*** ***˚C***	**E1**	23.51 ± 1.80^aA^	51.69 ± 1.37^aB^	65.94 ± 1.08^bC^	73.47 ± 1.29^bD^	78.60 ± 0.56^aE^
	**E2**	22.12 ± 1.41^aA^	52.63 ± 0.74^aB^	66.50 ± 5.11^bC^	73.05 ± 0.20^bCD^	77.27 ± 2.74^bD^
	**L1**	22.15 ± 2.82^aA^	39.50 ± 2.12^bB^	52.06 ± 1.32^dC^	66.01 ± 0.96^cD^	70.18 ± 2.86^cD^
	**L2**	22.66 ± 0.77^aA^	38.05 ± 0.77^bcB^	42.10 ± 0.56d^eC^	59.07 ± 0.74^dD^	65.29 ± 0.57^dE^
	**E1+L1**	22.99 ± 4.06^aA^	39.85 ± 2.61^bB^	51.35 ± 1.90^dC^	64.22 ± 0.53^cD^	70.70 ± 0.99^cE^
	**E1+L2**	23.46 ± 1.08^aA^	35.55 ± 0.77^cdB^	46.12 ± 0.80^efC^	54.91 ± 0.42^eD^	59.18 ± 0.73^eE^
	**E2+L1**	22.54 ± 0.80^aA^	40.30 ± 0.99^bB^	58.21 ± 1.54^cC^	68.95 ± 1.08^cD^	72.56 ± 0.79^cD^
	**E2+L2**	23.21 ± 5.09^aA^	33.29 ± 0.72^dB^	45.70 ± 0.56^efC^	48.64 ± 1.77^fC^	49.95 ± 0.64^fC^
	**BHT**	22.95 ± 2.34^aA^	33.35 ± 1.90^dB^	43.55 ± 1.48^fC^	46.77 ± 1.50^fCD^	49.75 ± 0.35^fD^
	**C**	22.78 ± 1.22^aA^	53.25 ± 1.06^aB^	76.83 ± 0.66^aC^	84.92 ± 0.27^aD^	88.11 ± 1.69^aE^

E_1_: 0.10% EEO, E_2_: 0.50% EEO, L_1_: 20 ppm lycopene, L_2_: 50 ppm lycopene, BHT: 0.02% BHT, C: Control.

Same uppercase and lowercase letters indicate no significant differences within a row and column, respectively (*p > *0.05).

**Table 2:** Free fatty acid values (%) of pasteurized creams treated by *Echinophora* *platyloba* DC. essential oil and lycopene during storage. Data are presented as mean ± standard deviation

	**Treatments**	** Day 0**	** Day 4**	**Day 7**	** Day 10**	** Day 14**
***4*** ***˚C***	**E1**	3.34 ± 0.45^aA^	3.84 ± 0.09^aAB^	4.16 ± 0.49^aAB^	4.70 ± 0.71^aAB^	4.96 ± 0.30^abB^
	**E2**	3.34 ± 0.51^aA^	3.89 ± 0.49^aAB^	4.11 ± 0.19^aAB^	4.70 ± 0.6^aAB^	4.96 ± 0.70^abB^
	**L1**	2.85 ± 0.28^aA^	3.27 ± 0.73^aAB^	3.63 ± 0.11^aAB^	3.70 ± 0.45^aAB^	4.30 ± 0.13^abB^
	**L2**	3.26 ± 0.80^aA^	3.63 ± 0.84^aAB^	3.97 ± 0.28^aAB^	4.70 ± 0.32^aAB^	4.45 ± 0.24^abB^
	**E1+L1**	3.13 ± 0.59^aA^	3.43 ± 0.31^aAB^	3.77 ± 0.40^aAB^	3.70 ± 0.25^aAB^	4.32 ± 0.40^abB^
	**E1+L2**	3.36 ± 0.15^aA^	3.61 ± 0.01^aAB^	3.94 ± 0.26^aABC^	4.70 ± 0.46^aABC^	4.30 ± 0.13^abC^
	**E2+L1**	2.94 ± 0.55^aA^	3.35 ± 0.40^aAB^	3.63 ± 0.20^aAB^	3.70 ± 0.17^aAB^	4.22 ± 0.98^abB^
	**E2+L2**	3.06 ± 0.20^aA^	3.35 ± 0.51^aAB^	3.50 ± 0.42^aAB^	3.70 ± 0.49^aAB^	4.08 ± 0.58^abB^
	**BHT**	2.97 ± 0.29^aA^	3.18 ± 0.12^aA^	3.25 ± 0.27^aA^	3.70 ± 0.38^aA^	3.57 ± 0.84^aA^
	**C**	3.35 ± 0.51^aA^	3.92 ± 0.20^aAB^	4.52 ± 0.12^aAB^	4.70 ± 0.80^aAB^	5.10 ± 0.57^bB^
***25*** ***˚C***	**E1**	3.31 ± 0.41^aA^	4.14 ± 0.12^aA^	6.12 ± 0.51^abB^	9.70 ± 0.53^abC^	11.08 ± 0.35^bD^
	**E2**	3.35 ± 0.06^aA^	4.09 ± 0.31^aA^	6.11 ± 0.09^abB^	8.70 ± 0.97^abcC^	11.05 ± 0.53^bD^
	**L1**	3.34 ± 0.37^aA^	3.83 ± 0.18^aA^	5.33 ± 0.45^abcB^	8.70 ± 0.66^abcC^	10.02 ± 0.05^cdD^
	**L2**	3.16 ± 0.28^aA^	3.75 ± 0.17^aA^	5.27 ± 0.62^abcB^	8.70 ± 0.44^abcC^	9.81 ± 0.27^cdD^
	**E1+L1**	3.05 ± 0.20^aA^	3.76 ± 0.56^aAB^	5.28 ± 0.15^abcB^	8.70 ± 0.65^abcC^	9.58 ± 0.41^cdC^
	**E1+L2**	3.41 ± 0.50^aA^	3.95 ± 0.46^aA^	5.16 ± 0.22^bcB^	7.70 ± 0.33^bcC^	8.86 ± 0.01^deD^
	**E2+L1**	3.94 ± 0.86^aA^	3.59 ± 0.54^aAB^	5.05 ± 0.82^bcB^	7.70 ± 0.29^bcC^	9.35 ± 0.42^cdC^
	**E2+L2**	2.97 ± 0.07^aA^	3.45 ± 0.42^aA^	4.90 ± 0.20^bcA^	7.70 ± 1.16^bcB^	8.77 ± 0.44^deB^
	**BHT**	3.44 ± 0.21^aA^	3.82 ± 0.18^aAB^	4.84 ± 0.18^cB^	6.70 ± 0.23^cC^	8.22 ± 0.33^eD^
	**C**	3.50 ± 0.44^aA^	4.45 ± 0.33^aA^	6.53 ± 0.51^aB^	10.70 ± 1.17^aC^	12.12 ± 0.13^aD^

E_1_: 0.10% EEO, E_2_: 0.50% EEO, L_1_: 20 ppm lycopene, L_2_: 50 ppm lycopene, BHT: 0.02% BHT, C: Control.

Same uppercase and lowercase letters indicate no significant differences within a row and column, respectively (*p > *0.05).

Table 3 presents amount of TBARS in experimental pasteurized creams during storage. All treated samples exhibited significantly much lower level of TBARS than the natural experimental creams (control). The increment of MDA values was much lower (*p* < 0.05) in BHT and samples containing lycopene, especially in combination with EEO. The lowest TBA value was related to 0.50% EEO+50 ppm lycopene (E_2_L_2_) treatments besides BHT.

**Fig. 2:**
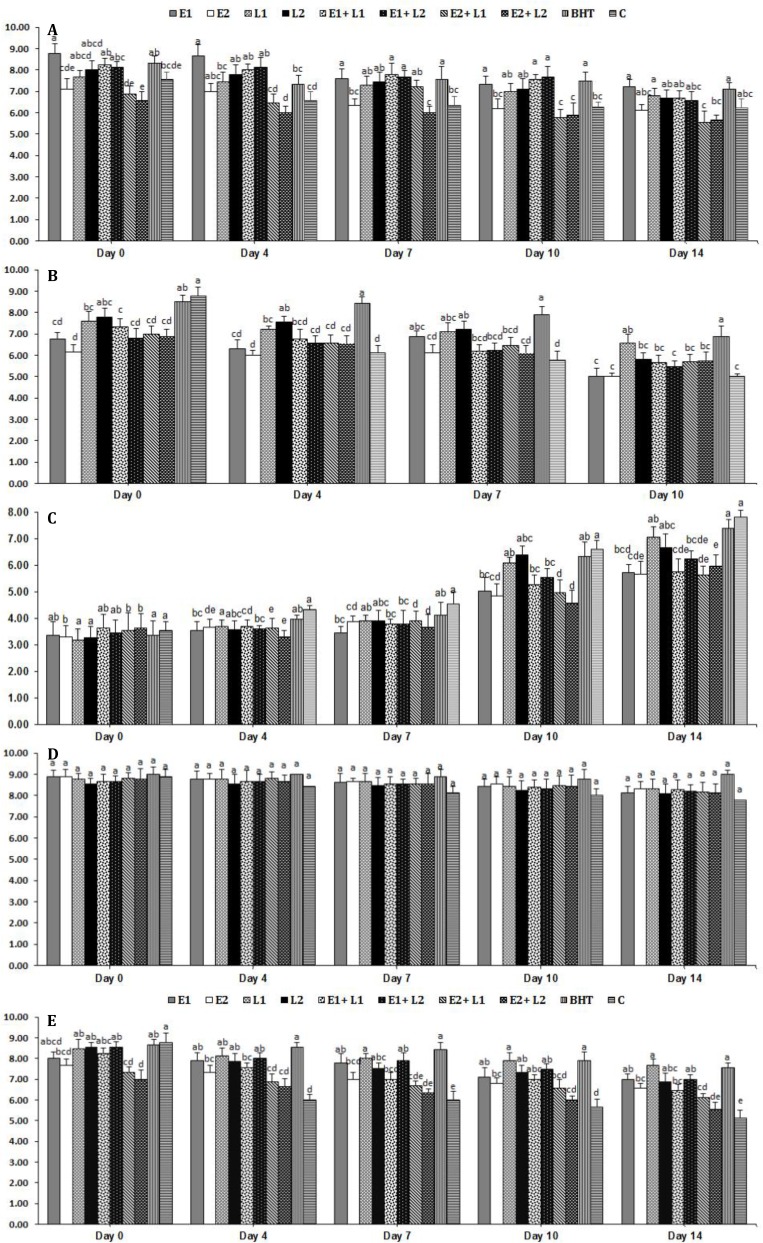
Sensory evaluation of pasteurized creams treated by *Echinophora platyloba* DC. essential oil and lycopene during storage at 4 ˚C. **A) **Odor,** B) **Taste,** and C) **Texture,** D) **Color, **E)** Overall acceptability of pasteurized creams treated by *Echinophora platyloba* DC. essential oil and lycopene during storage at 4 ˚C. Data are presented as mean ± standard deviation. Same letters indicate no significant differences (*p > *0.05).

**Sensory properties**. The effects of treatments and storage periods on the sensory properties of experimental pasteurized creams are shown in Figure 2. As it can be seen, sensory evaluation was performed only for samples stored at 4 ˚C. The lowest odor scores were observed in samples with high level of the EEO (0.50%), however they were in moderate acceptability range even at the end of storage time. Sensory evaluation for taste of samples was not performed on 14^th^ day of storage period due to probable spoilage of experimental creams.

Decrease of taste scores during storage in samples containing lycopene was lower than other treated creams and control. Sensory parameters indicated that generally, scores of the samples containing low level of the EEO (0.10%) were higher than those of the samples containing high level of the EEO (0.50%).

**Table 3:** Thiobarbituric acid reactive substances values (mg MDA per kg) of pasteurized creams treated by *Echinophora* *platyloba* DC. essential oil and lycopene during storage. Data are presented as mean ± standard deviation

	**Treatments**	** Day 0**	** Day 4**	** Day 7**	** Day 10**	** Day 14**
***4*** ***˚C***	**E1**	0.02 ± 0.00^aA^	0.03 ± 0.00^abB^	0.05 ± 0.00^abC^	0.06 ± 0.00^aD^	0.06 ± 0.00^aD^
	**E2**	0.02 ± 0.00^aA^	0.03 ± 0.00^abB^	0.05 ± 0.00^abcC^	0.06 ± 0.00^aD^	0.06 ± 0.00^aD^
	**L1**	0.02 ± 0.00^aA^	0.03 ± 0.00^abA^	0.04 ± 0.00^bcdB^	0.05 ± 0.00^bC^	0.05 ± 0.00^bC^
	**L2**	0.02 ± 0.00^aA^	0.03 ± 0.00^abA^	0.04 ± 0.00^dB^	0.05 ± 0.00^bcC^	0.04 ± 0.00^cdB^
	**E1+L1**	0.02 ± 0.00^aA^	0.03 ± 0.00^abA^	0.04 ± 0.01^bcdAB^	0.05 ± 0.00^bB^	0.05 ± 0.00^bcB^
	**E1+L2**	0.02 ± 0.00^aA^	0.03 ± 0.00^abAB^	0.03 ± 0.00^dBC^	0.04 ± 0.00^bcD^	0.04 ± 0.00^dCD^
	**E2+L1**	0.03 ± 0.00^aA^	0.03 ± 0.00^abA^	0.04 ± 0.00^bcdB^	0.05 ± 0.00^bC^	0.04 ± 0.00^bcBC^
	**E2+L2**	0.02 ± 0.00^aA^	0.03 ± 0.00^abAB^	0.03 ± 0.00^dABC^	0.04 ± 0.00^bcC^	0.04 ± 0.00^dBC^
	**BHT**	0.02 ± 0.00^aA^	0.02 ± 0.00^aA^	0.04 ± 0.00^dB^	0.04 ± 0.00^cB^	0.04 ± 0.00^dB^
	**C**	0.02 ± 0.00^aA^	0.03 ± 0.00^bB^	0.05 ± 0.00^aC^	0.07 ± 0.00^aD^	0.06 ± 0.00^aD^
***25*** ***˚C***	**E1**	0.02 ± 0.00^aA^	0.03 ± 0.00^abB^	0.05 ± 0.00^abC^	0.07 ± 0.00^abD^	0.07 ± 0.00^abD^
	**E2**	0.02 ± 0.00^aA^	0.04 ± 0.00^abB^	0.05 ± 0.00^abC^	0.00 ± 0.00^bD^	0.06 ± 0.00^bD^
	**L1**	0.02 ± 0.00^aA^	0.03 ± 0.00^abcB^	0.04 ± 0.00^bcC^	0.05 ± 0.00^cD^	0.05 ± 0.00^cD^
	**L2**	0.02 ± 0.00^aA^	0.03 ± 0.00^abcAB^	0.04 ± 0.00^bcBC^	0.05 ± 0.00^cdD^	0.05 ± 0.00^cD^
	**E1+L1**	0.02 ± 0.00^aA^	0.03 ± 0.00^abcB^	0.04 ± 0.00^cB^	0.05 ± 0.00^cdC^	0.05 ± 0.00^cC^
	**E1+L2**	0.02 ± 0.00^aA^	0.03 ± 0.00^abcB^	0.04 ± 0.00^bcC^	0.05 ± 0.00^deD^	0.04 ± 0.00^deCD^
	**E2+L1**	0.02 ± 0.00^aA^	0.03 ± 0.00^abcA^	0.04 ± 0.00^bcB^	0.05 ± 0.00^cdB^	0.05 ± 0.00^cdB^
	**E2+L2**	0.02 ± 0.00^aA^	0.03 ± 0.00^bcA^	0.04 ± 0.00^cB^	0.04 ± 0.00^eC^	0.04 ± 0.00^eBC^
	**BHT**	0.02 ± 0.00^aA^	0.03 ± 0.00^cA^	0.04 ± 0.00^cB^	0.04 ± 0.00^eB^	0.04 ± 0.00^eB^
	**C**	0.02 ± 0.00^aA^	0.04 ± 0.00^aB^	0.06 ± 0.00^aC^	0.07 ± 0.00^aD^	0.07 ± 0.00^aD^

E_1_: 0.10% EEO, E_2_: 0.50% EEO, L_1_: 20 ppm lycopene, L_2_: 50 ppm lycopene, BHT: 0.02% BHT, C: Control.

Same uppercase and lowercase letters indicate no significant differences within a row and column, respectively (*p > *0.05).

E1: 0.10% EEO, E2: 0.50% EEO, L1: 20 ppm lycopene, L2: 50 ppm lycopene, BHT: 0.02% BHT, C: Control.

## Discussion

Results of TAMB count of the studied samples during storage at 4 ˚C and 25 ˚C showed that samples containing EEO especially high level of the oil (0.50%) had stronger effect on reducing bacteria than samples of control group.

Psychrotrophic bacteria are defined as group of different bacterial species that are able to grow at 7 ˚C or less regard-less of their optimal temperature of growth.^3^ According to the results of this study, psychrotrophic count of samples with high concentration of EEO (0.5%) stored at 4 ˚C and 25 ˚C had significant difference with control samples. Samaržija* et al*. indicated that in approximately 25.00% of cases, psychrotrophic bacteria are the main causes of spoilage and reduced shelf-life of cream.^3^

The genus *Pseudomonas* are mentioned as the most often representatives of the gram negative population isolated from dairy products,^3^ which increase FFA by production of lipase and phospholipase enzymes.^19^ The findings of this study showed that *Pseudomonas* spp. count was not significantly different between experimental samples which can be resulted from lower sensitivity of gram negative bacteria to EEO as previously reported by the authors.^12^

To the best of authors’ knowledge, this is the first study representing the effect of EOs on TAMB count, *Pseudomonas* spp. or psychrotrophic count in pasteurized cream and the first study representing the effects of EEO in a food model but similar results have been reported for TAMB counts of butter containing *Thymus haussknechtii* and *Origanum acutidens* EOs^5^ and for psychrotrophic count changes in natural pasteurized double cream.^20^

Hydro-peroxides, known as the primary product of lipid oxidation, have a great impact on high fat dairy products shelf-life.^17^ Due to the unstable nature of peroxides, degradation products of aldehydes, ketones and alcohols are produced, which are mainly the cause of off-flavor.^21^ Results showed an increase in PV during storage in all of the investigated groups. Accordingly, samples treated with lycopene had stronger effect on delaying hydro-peroxide formation than samples treated with EEO and the effect of EEO and lycopene as combination was more considerable as alone. At the end of storage period, the rate of peroxide formation was lower than first days which could be a result of decomposition of hydro-peroxides into secondary products.^4^^,^^21^

Triglyceride hydrolysis produces FFA that is a quality indicator in fat-rich products.^11^ It has been shown that hydrolysis of lipids occurs even during refrigerated storage.^21^ In the present study, FFA count increased in samples stored at 4 ˚C and at room temperature during storage. Samples with combined application (E_2_L_2_) had lower FFA values compared to other treatments, indicating it decelerated lipid hydrolysis.^21^^,^^22^ Free fatty acids cause decrease in consumer acceptance of products due to causing deleterious effects on protein solubility, disagreeable flavors, discoloration, viscosity-related deterioration of the texture and more rapid oxidation to higher molecular weight lipids.^21^

As it was mentioned above, there was no report on properties of EEO or extracts in a food model and stability enhancement of pasteurized cream by any EOs as well, but similar results are obtained in a study by Kaur *et al*. on the effect of lycopene on PV and FFA values decrease during storage of butter samples containing 20 ppm lycopene.^11^ Similar to present study, increment of PV and FFA values with temperature heightening was also shown in a study on stability of natural butter.^6^

In this study, the TBA content of pasteurized cream was measured, which indicates degree of oxidation and rancidity by measuring concentration of secondary oxidation products mainly MDA.^22^ TBARS values showed fluctuation during storage (first increase followed by decrease on 10th day of storage time) in all of the studied groups, which may be due to the decomposition of the MDA and interactions between MDA and amino acids, proteins, glucose and other constituents during the storage of experimental creams.^21^ The TBARS results also indicated more effectiveness of lycopene than EEO in prevention of MDA formation. Similar pattern of fluctuation for TBARS values has been found by other researchers as well.^6^^,^^23^ The TBARS results in present study is consistent with the results obtained from butter and butter oil, which indicated that addition of EOs and extracts inhibited the formation of TBARS at 4 ˚C.^5^^,^^8^^,^^24^ The MDA over-generation in higher temperatures was also shown by Simsek which is in agreement with current results.^6^

The effects of treatments and storage periods on the sensory properties of experimental pasteurized creams were also assessed in this study. Accordingly, sensory scores decreased in all samples due to microbial and chemical changes during storage. The decrease in scores was less in samples with EEO or lycopene than control and all scores were acceptable by panelist during storage. Scores for color attribute of cream samples were similar within storage time but the scores of odor, taste, texture, and overall acceptability for control and treated cream samples varied. Samples with low concentration of EEO had the highest odor scores indicating positive effect of the EEO addition on this characteristic of pasteurized creams. Results of sensory evaluation for taste and texture attributes showed the negative effect of the EEO and positive effect of lycopene on these scores of experimental cream samples even though the minimum scores of taste and texture were in moderate acceptability range. At the early days of storage, strong odor of the EEO, especially at higher levels, resulted in reduction in overall acceptability scores and therefore, the scores were lower in entire storage time in samples containing high concentration of the EEO (0.50%). These results are similar to other works mentioned that addition of EOs or lycopene could enhance shelf-life and sensory parameter scores in experimental butters.^11^^,^^25^

In conclusion, the results of microbial and chemical stability analyses of pasteurized creams revealed that the addition of EEO and lycopene caused shelf-life enhance-ment of experimental pasteurized creams without having adverse effect on their quality. Besides, notable effects were observed in analyses of microbial and chemical stability of creams followed by combined addition of lycopene and the EEO, especially in samples containing high levels of lycopene (50 ppm) and the oil (0.50%). The data achieved by sensory evaluation revealed a good acceptability of pasteurized creams containing EEO and lycopene. Therefore, based on microbial, chemical and sensorial properties of treated pasteurized creams by EEO and lycopene, they can be practically applied in stability of high fat products especially in butter and creams and by means of such functional foods, the producers and consumers will avail the benefits of shelf-life extended products as well as natural antioxidants and antimicrobials.
